# *Clonostachys rosea*: Production by Submerged Culture and Bioactivity Against *Sclerotinia sclerotiorum* and *Bemisia tabaci*

**DOI:** 10.3389/fmicb.2022.851000

**Published:** 2022-05-06

**Authors:** Gabriel Moura Mascarin, Ana Vitória Reina da Silva, Thiago Pereira da Silva, Nilce Naomi Kobori, Marcelo Augusto Boechat Morandi, Wagner Bettiol

**Affiliations:** ^1^Brazilian Agricultural Research Corporation, Embrapa Environment, Jaguariúna, Brazil; ^2^Independent Researcher, Jaguariúna, Brazil

**Keywords:** biological control, submerged liquid fermentation (SLF), solid-state fermentation (SSF), whitefly (*Bemisia tabaci*), white mold (*Sclerotinia sclerotiorum*)

## Abstract

Among the prospective biocontrol agents, the saprophytic filamentous fungus *Clonostachys rosea* is an excellent necrotrophic mycoparasite of numerous plant pathogenic fungi. However, its commercial development has been hampered by mass production difficulties during solid-state fermentation. Conversely, the submerged liquid fermentation shortens the cultivation time while increasing yields of fungal propagules. However, this method has been overlooked for *C. rosea*. In this work, we investigated the impact of liquid pre-culture inoculum on the spore production by the two-stage fermentation process using rice grains in comparison to the traditional solid-state fermentation. In parallel, we studied the submerged cultivation of *C. rosea* by manipulating carbon-to-nitrogen (C:N) ratio and nitrogen source, with the further optimization of spore production in a benchtop bioreactor. Additional bioassays included assessing the bioactivity of water-dispersible microgranules (that contained a submerged conidia) against the whitefly (*Bemisia tabaci* biotype B) and *Sclerotinia sclerotiorum* (causal agent of the white mold). Our results showed a maximum concentration of 1.1 × 10^9^ conidia/g-dry-matter after 7 days of cultivation by two-stage fermentation process. The liquid fermentation yielded 1.4 × 10^9^ submerged conidia/ml after 7 days using a medium with a 50:1 C:N ratio, and it also induced the production of microsclerotia (MS) up to 1.35 × 10^4^/ml within 6 days with 10:1 C:N ratio; both media were supplemented with dextrose monohydrate and soybean meal. The fermentation batches carried out in a benchtop bioreactor with medium 50:1 C:N ratio and amended with soybean meal rendered a production peak on the fourth day, corresponding to 1.11 × 10^9^ conidia/ml and 4.35 × 10^8^ colony forming units (CFU)/ml. Following air-drying, the conidia production from air-dried microgranules of *C. rosea* biomass was estimated at 3.4 × 10^10^ conidia/g of formulated product upon re-hydration for 7 days. Both submerged conidia and MS of *C. rosea* inhibited 100% germination of *S. sclerotiorum* sclerotia by direct parasitism. The air-dried submerged conidia exhibited a suppressive activity on sclerotia (88% mycoparasitism) and early whitefly nymphs (76.2% mortality) that rendered LC_50_ values of 3.2 × 10^4^ CFU/g soil and 1.5 × 10^7^ CFU/ml, respectively. Therefore, the submerged liquid culture of *C. rosea* may offer a feasible and cost-effective method for its large-scale production, alleviating critical constraints to their commercial use while providing an additional tool for management of *B. tabaci* and *S. sclerotiorum*.

## Graphical Abstract

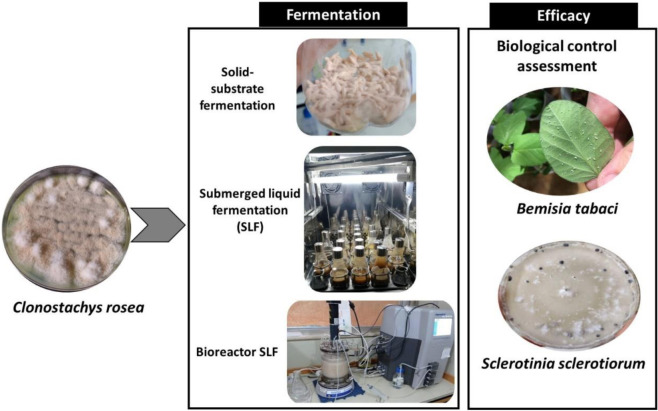

## HIGHLIGHTS

- Fermentation methods were compared for *Clonostachys rosea* propagule production.
- Submerged liquid fermentation was optimized by modifying nitrogen sources and C:N ratios.
- First evidence of *C. rosea* microsclerotia formation in liquid culture.
- Production of submerged spores was enhanced when cultivated in a benchtop bioreactor.
- Submerged spores survived well after the drying process.
- The *C. rosea* bioactivity against sclerotia of *Sclerotinia sclerotiorum* and whitefly nymphs was demonstrated.

## Introduction

The biological control in agriculture can be defined as the use of an organism to reduce the population density of another organism that causes losses in agricultural production, such as pests, diseases, and invasive plants (van Lenteren et al., 2020). One of the types of the biological control is augmentative, in which antagonists, entomopathogens, parasitoids, and predators are extensively applied in agriculture. Carrying out a large-scale multiplication of the biocontrol agents is essential for the development of this technique, and this phase represents a major limiting factor for the growth and the implementation of a massive biological control program. An inoculative or inundative biological control against a broad range of targeted pests make use of hyphae, aerial and submerged conidia, blastospores, chlamydospores, and microsclerotia (MS), which are commercial propagules produced by ascomycete filamentous fungi in different culture media (Wraight et al., 2001; de Faria and Wraight, 2007; Mascarin and Jaronski, 2016).

The fungi of the genus *Clonostachys* (Ascomycota: Bionectriaceae) are widespread as soil inhabitants, plant decomposers, and endophytes commonly found in tropical and subtropical regions (Schroers, 2001). Due to the ability to suppress the sporulation of plant pathogenic fungi (mycoparasitic lifestyle), colonize senescent and dead tissues, promote plant growth, and induce plant resistance, *Clonostachys* has gained momentum as a multifunctional biocontrol agent (Sutton et al., 1997; Morandi et al., 2001; Nobre et al., 2005; Mouekouba et al., 2014). Particularly, *Clonostachys rosea* (syn. *Gliocladium roseum*) stands out in importance not only as a necrotrophic mycoparasite of several plant pathogenic fungi, but also parasitizing insects and plant pathogenic nematodes (Sutton et al., 1997; Anwar et al., 2018; Carvalho et al., 2018; Sun et al., 2020).

The commercial development of the fungal biopesticides is critically dependent on the amenability and the ease of mass production of the fungal strain on a large-scale setting, and this requires a cost-effective media and cultivation process to be viable. Accordingly, the mass production of fungal biological control agents relies on solid, liquid or biphasic fermentation processes (Mascarin and Jaronski, 2016). The solid-state fermentation method consists in the use of cereal grains as the main growth substrate, while the biphasic or two-stage fermentation process proposes a first step of growth by liquid culture which is subsequently used as inoculum source to attain the production of aerial conidia grown on the solid substrate. The solid-substrate fermentation presents bottlenecks, such as high costs of cereal-based substrates, intense demand for the labor, greater chance of contamination, automation deficiencies, long fermentation periods, and the lack of control of both nutritional and environmental conditions (Jackson, 1997; Mascarin et al., 2015; Santos P. S. et al., 2021).

To date, there have been only a few commercial products based on *C. rosea* available worldwide, such as Vectorite^®^ and Endofine^®^ in Canada (Canadian Patent Application, 2007; Bettiol et al., 2021) and Kamoi^®^ in Brazil (Kamoi, 2021). The industrial production of these products is carried out on solid substrates made of cereal grains. In contrast, the submerged culture process offers several advantages over the traditional solid-state fermentation method, since the former provides a cost-effective and more efficient production system due to the reduced cultivation time and increased economic and productivity gains (Jackson, 2000). Besides the possibility of controlling the nutrients in the media, such as vitamins, salts, carbon, and nitrogen sources, this process also enables easiness and flexibility in manipulating the physical environment during the fermentation process, including aeration rate, dissolved oxygen, pH, temperature, osmotic pressure, and foaming. Coupled with these advantages, submerged culture allows incomparable versatility for the production of propagules of interest, such as submerged conidia, blastospores, mycelia, chlamydospores, and microsclerotia (Jackson et al., 2010; Mascarin and Jaronski, 2016).

With respect to tackling the control of potential target hosts by *C. rosea*, the whitefly *Bemisia tabaci* (Gennadius, 1889) (Hemiptera: Aleyrodidae) and the white mold disease caused by the fungus *Sclerotinia sclerotiorum* (Lib.) de Bary (Ascomycota: Sclerotiniaceae) are the two most globally destructive and yield-limiting pest and plant pathogen, respectively, affecting several crops of economic importance, including soybean, cotton, bean, tomato, potato, canola, and sunflower (Perring et al., 2018; O’Sullivan et al., 2021). Collectively, these two noxious organisms are responsible for the multi-billion losses every year in soybean producing regions, with estimated economic losses up to US$1.2 billion in the United States (Allen et al., 2017) and US$1.47 billion in Brazil (Lehner et al., 2016) due to the white mold disease, while the whiteflies can cause around US$1.0 billion yield losses annually to many crops in Brazil (Oliveira et al., 2013). To counteract the overuse of chemical pesticides associated with recurrent selection of resistant *B. tabaci* and *S. sclerotiorum* strains, the fungal biocontrol agents are considered environmentally friendly alternatives for sustainable integrated management of these targets. However, the dual bioactivity of *C. rosea* using its submerged propagules obtained by liquid culture has not been investigated so far against these target insect pest and plant pathogen.

This study represents the first attempt to determine the impact of inoculum type on conidial production through solid-state fermentation, and to investigate the nutritional requirements in the growth medium for the optimization of submerged liquid fermentation of *C. rosea.* The different inoculum types produced in the above-mentioned fermentation systems were evaluated in terms of their ability in controlling *B. tabaci* nymphs in common beans (*Phaseolus vulgaris* L.) and *S. sclerotiorum* sclerotia. This study is aimed to (i) assess the impact of the types of inoculum on the production of aerial conidia of *C. rosea* grown on rice grains through a biphasic fermentation, (ii) optimize the nutritional environment by altering C:N ratio and nitrogen source in the submerged culture of *C. rosea*, and (iii) evaluate the effectiveness of liquid-grown *C. rosea* propagules against *S. sclerotiorum* and *B. tabaci* under laboratory conditions.

## Materials and Methods

### Fungal Strains and Culture Maintenance

The *C. rosea* strain CMAA1284 (GenBank accession MG489966) used in these studies was isolated from rose crops in Viçosa, Minas Gerais State, Brazil; the *S. sclerotiorum* strain CMAA1105 (GenBank accession OM348513) used was isolated from soybean. All fungi used in this research were deposited at Embrapa Environment Collection of Microorganisms of Agricultural and Environmental Importance (CMAA). The fungi were preserved by means of fragments, 5 mm in diameter, of fully-grown colonies, added in cryovials filled with 1.5 ml of sterile 20% glycerol solution (v/v), and stored at −40°C. From the cryotubes, the microorganisms were routinely transferred to Petri dishes (90 × 15 mm, Pleion^®^) that contained 20 ml of potato–dextrose–agar (PDA, Difco^®^) medium and incubated in growth chamber for 14 days at 25 ± 1°C and with 12:12 h photoperiod. The fungal strains have been registered under the Brazilian genetic heritage – Sisgen – protocol A00AFAF.

### Biphasic Fermentation on Rice Grains

To study the biphasic fermentation of *C. rosea*, 300 g of parboiled rice were moistened with 600 ml of distilled water for 1 h. Then, the excess water was removed and 15 g of the wet rice (initial moisture content of 39% w/w) were distributed in each Erlenmeyer flask (125 ml, baffled type, Exom^®^, São Paulo, SP, Brazil), sealed with hydrophobic cotton plugs and then covered by aluminum foil for autoclaving for 20 min at 121°C. After autoclaving, the aluminum foil was removed to allow the gas exchange during the fermentation process. This trial included two treatments, whose objective was to investigate the influence of two types of inoculum on the conidial production by a solid-state fermentation using the parboiled rice grains. Treatment 1 consisted of the suspension containing submerged conidia produced by liquid culture (hereafter referred to as liquid pre-culture), and treatment 2 was assigned to the suspension of aerial conidia obtained from sporulated cultures grown on PDA for 14 days and then suspended in 10 ml of sterile 0.04% polysorbate solution (Tween 80^®^), in order to obtain a inoculum adjusted to 1 × 10^7^ conidia/ml for further inoculation of 90 ml of liquid medium with 10 ml (10% v/v) of this spore suspension. Briefly, the pre-culture of *C. rosea* was obtained with a liquid medium consisting of dextrose monohydrate and yeast extract with a 50:1 C:N ratio and 36 g/L of carbon content (total volume of 100 ml in 250-ml baffled Erlenmeyer flask) grown for 4 days (the pre-culture is described in Table 1). These pre-cultures were quantified based solely on submerged conidia with barely hyphal fragments in the final inoculum concentration. The flasks that contained 15 g of sterile moistened rice mass were inoculated with 1.5 ml (10% inoculum volume) of each suspension of *C. rosea* adjusted to 5 × 10^6^ aerial or submerged conidia/ml to deliver a final concentration of 5 × 10^5^ aerial or submerged conidia/g rice (on wet weight basis). The flasks were vigorously hand-shaken for 1 min to homogenize the inoculum throughout the rice mass. The inoculated rice cultures were kept static (without agitation) in a growth chamber set to 25 ± 1°C with a 12:12 h photoperiod for 7 days. After this period, fully sporulated fungus-colonized rice mass was suspended in 50 ml of surfactant solution (0.05% Break Thru^®^ MSO, Evonik^®^, Essen, Germany), placed in a rotary incubator shaker with diameter orbit (or throw) of 28 mm (SL-223–5, Solab^®^, Piracicaba, SP, Brazil) and agitation speed of 248 rpm, with constant temperature at 28 ± 1°C for 30 min. The flasks were subsequently taken to ultrasound water bath for 5 min to promote the detachment of conidia from the substrate (see the protocol details in Supplementary Figure S1). After these steps, a serial dilution was performed and the conidia were counted in a Neubauer (hemocytometer) chamber under a light microscope (Leica^®^ MD250, Germany) with 400 × magnification. The results were expressed in conidia/g-dry-matter (gDM) of rice previously considering the initial rice moisture (average of 39% w/w). The experiment was carried out in a completely randomized design (CRD) involving two treatments with six replicates each, obtaining *n* = 12 replicates for each treatment, since the entire experiment was repeated twice on different occasions using a new fungal inoculum.

**TABLE 1:** Composition of liquid culture media used in submerged liquid fermentation of *C. rosea* (strain CMAA1284).

Ingredients	Pre-culture	1	2	3	4	5	6	7	8
	Medium								
C:N ratio	50:1	50:1	50:1	50:1	50:1	10:1	10:1	10:1	10:1
pH initial	6	6	6	6	6	6	6	6	6
Inoculum (1 × 10^7^ conidia/ml) (ml)	10	10	10	10	10	10	10	10	10
Basal medium (ml)^†^	50	50	50	50	50	50	50	50	50
Dextrose 25% (ml)	37.2	37.2	36.4	36	36.4	21.2	18	15.4	17.7
Distilled water (ml)	2.1	2.1	3.6	4	3.6	15.5	22	24.6	22.4
Yeast extract (g)	0.7	0.7				3.30			
Cottonseed flour (g)			0.76				3.83		
Soybean meal (g)				0.84				4.25	
Corn bran (g)					0.77				3.90
Total volume (ml)	100	100	100	100	100	100	100	100	100

*^†^The composition of 1 L of basal medium was prepared with KH_2_PO_4_ 2.0 g, CaCl_2_.H_2_O 0.4 g, MgSO_4_.7H_2_O 0.3 g, FeSO_4_.7H_2_O 0.05 g, CoCl_2_.6H_2_O 37 mg, MnSO_4_.H_2_O 16 mg, ZnSO_4_.7H_2_O 14 mg, and vitamins including thiamine, riboflavin, calcium pantothenate, niacin, pyridoxamine, thioctic acid 500 μg each, and folic acid, biotin, vitamin B_12_ 50 μg each (Jackson et al., 1997).*

### Submerged Liquid Fermentation to Selection of Nitrogen Sources

In this study, the protein sources studied in the nutritional composition of the culture medium were as follows: (1) Yeast extract (10.4% nitrogen and 40% carbon; Acumedia/NEOGEN^®^, Lansing, MI, United States), (2)- cottonseed flour (9.4% nitrogen and 40% carbon; Pharmamedia^®^, ADM Co., Decatur, IL, United States), (3) corn bran (9.3% nitrogen and 40% carbon; Protenose^®^, Ingredion, Mogi Guaçu, SP, Brazil), and (4) soybean meal (8.5% nitrogen and 40% carbon; Baker’s soyflour^®^, ADM Co., Decatur, IL, United States). All sources initially contained 36-g carbon/L, had initial pH value of 6.0 and a C:N ratio of 10:1 for the MS formation and a preferred C:N ratio of 50:1 for the conidia formation. The 14-day-old sporulated PDA cultures of *C. rosea* were used to prepare liquid pre-cultures as described in the previous section. A sterile stock solution of 25% (w/v) dextrose monohydrate (35% carbon; Cerelose^®^, Ingredion^®^, Mogi Guaçu, SP, Brazil) was used in all fermentation studies as the main carbon source (Table 1). The experiment was carried out in a CRD including eight treatments with three replicate flasks each, and repeated three times on different dates.

All liquid media (100 ml per shake flask) tested in this fermentation study were inoculated with a standard spore suspension (10% inoculum volume) obtained from the liquid pre-culture. Briefly, the inoculation procedure applied to all liquid media was performed by adding 10 ml of the suspension (pre-culture) that contained 1 × 10^7^ conidia/ml into baffled Erlenmeyer flasks (250 mL) with stainless steel caps and filled with 90 ml of medium. These liquid cultures were incubated in an orbital rotary shaker (248 rpm) at 28 ± 1°C and 12:12 h photoperiod for 7 days. The counting of spores and MS were carried out from the second to the seventh day of the fermentation. To determine the concentration of the submerged conidia, a 1-ml aliquot of liquid culture was removed from the flasks and successively diluted for the counting in a Neubauer chamber. The quantification of MS was performed on a glass slide covered with a cover slip (24 × 50 mm). The fermented broth underwent a 10^–1^ dilution and 100 μL was added directly to the slide. Only MS with at least a diameter greater than 50 μm were computed, while the submerged conidia ranged in size from 3.86 to 8.21 μm (mean ± SE: 5.93 ± 0.22 μm) in length and from 2.89 to 4.71 μm (mean ± SE: 3.76 ± 0.14 μm) in width. The results were expressed in submerged conidia/ml and MS/ml.

The determination of the viable propagules was performed based on the counting of the colony forming units (CFU) in Petri dishes (90 × 15 mm) that contained 20 ml of PDA amended with 0.01% (v/v) of Triton X-100^®^ (Synth^®^) and 0.001% (w/v) of chloramphenicol (Sigma^®^). After the serial dilution of the samples obtained from the liquid cultures, an aliquot of 50 μL of the suspension of *C. rosea* from the 10^–7^ dilution was transferred to the medium. The plates were incubated in a growth chamber at 25 ± 2°C with a photoperiod of 12:12 h, and the number of CFU was computed after 4 days. To carry out the drying of the biomass produced, on the seventh day of the fermentation, the entire volume of the liquid culture was mixed with 5% of diatomite (Diatom^®^ M45, Brazil) and filtered (slow filtration with filter paper; pore size <12 μm, 80 g/m^2^; Whatman^®^) through a Büchner funnel coupled to a Kitasato to dewater the biomass-diatomite mixture for the next step involving air drying. The drying was carried out in a horizontal tray chamber with controlled airflow (relative humidity of the purged air: 18–50%) for 15 h at 22 ± 2°C until reaching the final moisture content less than 5% (w/w) (Kobori et al., 2015). After drying, the materials were ground to fine granules (<2 mm) as also referred here as air-dried microgranules (see details in Supplementary Figure S2).

### Bioassay of *Clonostachys rosea* vs. *Sclerotinia sclerotiorum*

The study of *C. rosea* parasitism using different propagules on *S. sclerotiorum* sclerotia was carried out with 24 Petri dishes (90 × 15 mm) that contained 30 g of autoclaved dry soil in each plate, with moisture adjusted to 100% of field capacity, using sterile distilled water. The dark mature sclerotia of *Sclerotinia* were collected from 25-day-old PDA cultures and used in bioassays. After the distribution of 12 sclerotia per plate, 10.5 ml of *C. rosea* suspension that contained 16 μl of surfactant solution (0.02% of Break-thru MSO, Evonik^®^, Essen, Germany) were evenly applied to the soil in each plate. For control, 10.5 ml of distilled water was applied in the same way as mentioned above. The plates were incubated for 14 days in a growth chamber at 25 ± 1°C with 12:12 h photoperiod. After that period, all sclerotia were removed from the soil and then superficially disinfested with 70% ethanol (1 min), 2% sodium hypochlorite (NaOCl) (1 min), and rinsed three times in sterile distilled water prior to plating them on a selective medium. Twelve surface-sterilized sclerotia were transferred to Neon-S medium [1 L of water; 40 g of PDA (Difco^®^), 50 mg of bromophenol blue, 50 mg of chloramphenicol, and 50 mg of free acid 2,4-D)] per plate (20 ml medium, 90 × 15 mm), and then incubated for 7 days to evaluate viability (Ferraz et al., 2011). The viability was carried out by observing the biochemically induced color change in the Neon-S medium due to the pathogen germination, i.e., viable or germinated sclerotia change the color of the medium from purple to yellowish due to the production of oxalic acid (Napoleão et al., 2006; Ferraz et al., 2011). In addition, we also determined the *C. rosea* presence by assessing its outgrowth on sclerotia, as a surrogate to confirm the ability of this bioagent to penetrate, parasite, and kill the target pathogen structure (Rodríguez et al., 2011). In this bioassay, we tested the following four treatments: Control with distilled water, suspension of *C. rosea* conidia from solid fermentation on rice, suspension of *C. rosea* conidia from liquid fermentation (medium M3 in Table 1), and suspension of *C. rosea* MS from liquid fermentation (medium M7 in Table 1). The inoculum density was standardized to 1 × 10^6^ CFU/g of sterile dry soil, which was obtained with air-dried granules that contained 6 × 10^8^ CFU/g, except for the treatment with MS, which delivered 2.5 × 10^5^ MS/g of dry soil. The assays were performed twice on different dates, with six replicates for each treatment. An illustrative flowchart of this experimental procedure is described in Supplementary Figure S3.

### Bench-Scale Bioreactor Fermentation of *Clonostachys rosea*

The performance of submerged liquid fermentation process of *C. rosea* for mass production of submerged conidia was assessed and validated using a 3-L laboratory benchtop bioreactor (New Brunswick™ BioFlo/CelliGen^®^ 115, Eppendorf^®^, New Brunswick, NY, United States) with the medium coded as M3 (Table 1): 50:1 C:N ratio, 36 g carbon/L, pH 6.0, initial inoculum density of 5 × 10^6^ submerged conidia/ml, 9.1% (w/v) dextrose monohydrate and 0.84% (w/v) soybean meal (Bakers souflour^®^). The liquid pre-culture was the same as previously described (Table 1). The medium was inoculated with a 4-day-old liquid pre-culture to deliver a final concentration of 5 × 10^6^ conidia/ml using 180 ml (10% v/v) of a suspension prepared with sterile distilled water and adjusted to 5 × 10^7^ submerged conidia/ml, considering a total working volume of 1.8 L. The initial pH of the medium was adjusted to 6.0 without any control during the cultivation process. The initial parameters after inoculation were: agitation speed of 400 rpm, gas-flow of 1.0 L/min (0.56 vvm), and temperature of 28 ± 0.5°C. The mechanical agitation was propelled with two Rushton (flat blade) impellers. Antifoam solution at 0.05% (v/v) (Break-thru^®^ AF 9903, Evonik Operations GmbH, Essen, Germany) was pumped into the culture broth whenever needed to prevent foaming. Temperature, dissolved oxygen level (DO), gas-flow and agitation speed were monitored across time, and culture samples were taken every 24 h until the fourth day to determine the submerged spore concentration, the CFU and the pH. Then, both the agitation speed and the gas-flow (purged with filtered atmospheric air inlet) were altered during the fermentation process as a means to avoid DO dropping below 15%. This fermentation process was carried out in batch cultures and independently repeated on six different dates.

The 4-day-old culture broth that contained submerged conidia and mycelium (without any microsclerotium biomass) was harvested, centrifuged (10,000 rpm for 20 min at 10°C), and the resulting fresh biomass (20.0 g) was evenly mixed with 37.0 g (61.6% w/w) of diatomaceous earth (DE) [Diatom^®^ M45; bulk density, 280–360 g/L; fine powder < 0.12 mm (or < 150 mesh); Diatom Mineração Ltda., Mogi das Cruzes, SP, Brazil] and 3.0 g (5% w/w) of organosilicon-based dispersant (Break-thru SD260, Evonik Operations GmbH, Essen, Germany) to a final weight of 60-g formulated fungal biomass. The drying process followed the procedure described in Kobori et al. (2015) and is the same in the section “Submerged liquid fermentation to selection of nitrogen sources.” After 14–16 h of air-drying, the air-dried microgranular formulation held a final moisture content in the range of 2.6 – 3.6% (w/w) from different bioreactor fermentation batches (*n* = 6). After that, this fungal preparation was vacuum packed with a 5-layer barrier bag made of nylon-poly plastic (15 × 22 cm, Equapack Embalagens, São Paulo, SP, Brazil) and then cold stored at 4–6°C until use in bioassays to further evaluate the efficacy against sclerotia of *S. sclerotiorum* and whitefly nymphs. Before each bioassay, the air-dried submerged conidia had their viability checked on PDA medium, and it was expressed in CFU/g. For the assessment of myceliogenic germination, a sample of 0.03 g of each formulation batch was sprinkled over the surface of 2% (w/v) agar-water medium poured in Petri plate (90 × 15 mm) and incubated for 24 h at 25 ± 1°C and 12:12 h photoperiod. The percent viability was determined by randomly counting 100 fungal microgranules under a stereomicroscope at 40× magnification and deemed germinated in the presence of hyphal outgrowth. After an additional 6 days of incubation, the sporulated microgranules exhibiting profuse fungal outgrowth and conidiation were harvested using sterile 0.04% Tween 80^®^ solution and then serially diluted to proceed with counts on a Neubauer chamber in order to compute the spore production per gram of this air-dried microgranular formulation (Supplementary Figure S2).

### Bioefficacy of Air-Dried Microgranular Formulation Against Sclerotia of *Sclerotinia sclerotiorum*

The air-dried microgranular formulation that contained mainly submerged conidia of *C. rosea* retained viability of 6 × 10^8^ CFU/g. The inoculum loads tested to deliver active viable propagules to the soil were 1 × 10^4^, 1 × 10^5^, and 1 × 10^6^ CFU/g soil. The whole bioassay was repeated twice on different occasions with a total of 12 replicates per treatment. The methodology was the same as described before in the section “Bioassay of *C. rosea vs. S. sclerotiorum.*”

### Bioefficacy Against Whitefly Nymphs

The whitefly colony of *B. tabaci* biotype B or Middle East–Asia Minor I (MEAM1) was reared on cabbage (*Brassica oleracea* L., cv. Manteiga, TopSeed^®^, SP, Brazil) and on Jack bean (*Canavalia ensiformis* L., Piraí Sementes Ltda., Piracicaba, SP, Brazil) in a screenhouse under natural environmental conditions. The bean plants cv. Pérola were grown in the potted-soil inside a screenhouse and infested with the whitefly adults for 24 h to allow oviposition underside the leaves. After laying their eggs, the whitefly adults were removed from the bean plants by blowing them away (Silva et al., 2019). After approximately 12–14 days, the whitefly nymphs had reached the first to the second instars. The air-dried submerged conidia produced by the liquid culture in the bioreactor were tested against the first to the second instar nymphs to assess their pathogenicity and virulence using a concentration-mortality bioassay. The four concentrations of air-dried microgranular formulation of *C. rosea* (2.2 × 10^9^ CFU/g) prepared with 0.04% Tween 80^®^ were tested at: 5 × 10^6^, 1 × 10^7^, 5 × 10^7^, and 1 × 10^8^ CFU/ml. The sterile aqueous 0.04% Tween 80^®^ solution was sprayed as control. The potted bean plants bearing leaves infested with the whitefly nymphs were individually sprayed with a hand-held dual-gravity airbrush assembled to a benchtop spray-tower loaded with 300 μL of each spore concentration (Silva et al., 2019). After spraying, these potted bean plants were incubated in a growth chamber set to 26 ± 2°C and 12:12 h photoperiod with a data logger (HOBO^®^ U12-012, Sigma Sensors, São José dos Campos, SP, Brazil) to track-record the hourly temperature and the relative humidity inside this chamber throughout the experimental course. According to the data logger recordings during the 7 days of incubation, the mean temperature and relative humidity were 26.8°C (25.7–27.8°C) and 84.2% (30.7–96.4%), respectively. The number of nymphs per leaf varied greatly and ranged from 4 to 34, with the majority above 10 nymphs per leaf. The bioassay was repeated twice on different dates and each treatment had a total of seven replicates. The dead and live nymphs were recorded after 5 and 7 days of incubation. Dead nymphs, exhibiting typical symptoms of mycosis by *C. rosea*, appeared shriveled, dehydrated, and generally with orange-like or pinkish color.

### Data Analysis

The production data of aerial conidia, submerged conidia, and MS were fitted to generalized linear models with negative binomial distribution with or without fixed effects interaction in the linear predictor. When a significant effect was detected, the treatment means were compared by Tukey HSD at *p* < 0.05.

The whitefly mortality proportion, obtained across increased concentrations of *C. rosea* submerged conidia, was fitted to a two-parameter log–logistic model with binomial distribution using the “drc package” in R (Ritz et al., 2015). In addition, the median lethal concentrations for the mortality rates recorded on days 5 and 7 post-spraying were estimated and then compared by the *z*-score test and slopes by the *t*-Student test. Similarly, the data generated on proportion of non-viable or parasitized sclerotia of *S. sclerotiorum* after exposure to different concentrations of spore-treatments with *C. rosea* were fitted to the same model as described for the whitefly mortality, including the concentration of *C. rosea* inoculum as the fixed effect in the linear predictor. An estimated LC_50_ of *C. rosea* submerged conidia was also computed based on the antagonism exerted over sclerotia.

All statistical analyses were performed in the R statistical software environment (R Core Team, 2015^1^).

## Results

### Biphasic Fermentation on Rice Grains

**FIGURE 1:**
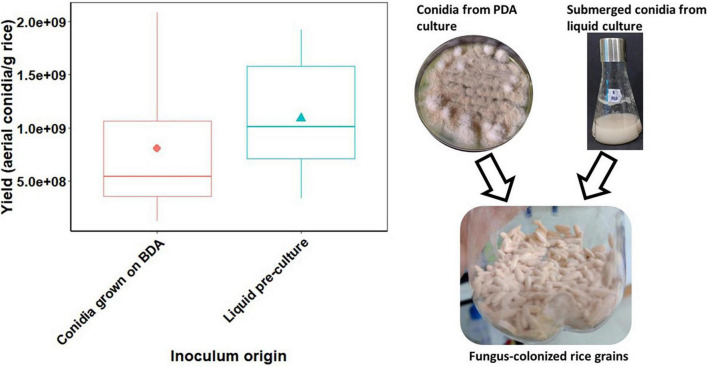
Impact of inoculum type (PDA-grown aerial conidia and conidia-grown in liquid culture) on the yield of aerial conidia of *Clonostachys rosea* (strain CMAA1284) after 7 days of cultivation on sterile parboiled rice grains.

The highest *C. rosea* conidial concentration was observed after 7 days of cultivation in Erlenmeyer flasks filled with autoclaved wet rice grains and inoculated with submerged conidia produced by liquid culture. Under these conditions, *C. rosea* produced 1.1 × 10^9^ conidia/gDM. The inoculum of the suspension made with aerial conidia grown on PDA medium gave an average of 8.1 × 10^8^ conidia/gDM. Nonetheless, there was no significant difference in conidial yield on rice grains due to the inoculum origin (χ^2^ = 1.26, *p* = 0.26), which means that solid-state fermentation of *C. rosea* on rice grains can be performed with any inoculum type of *C. rosea* either grown on PDA or in liquid medium (Figure 1).

### Liquid Fermentation of *Clonostachys rosea* in Different N Sources and C:N Ratios

**FIGURE 2:**
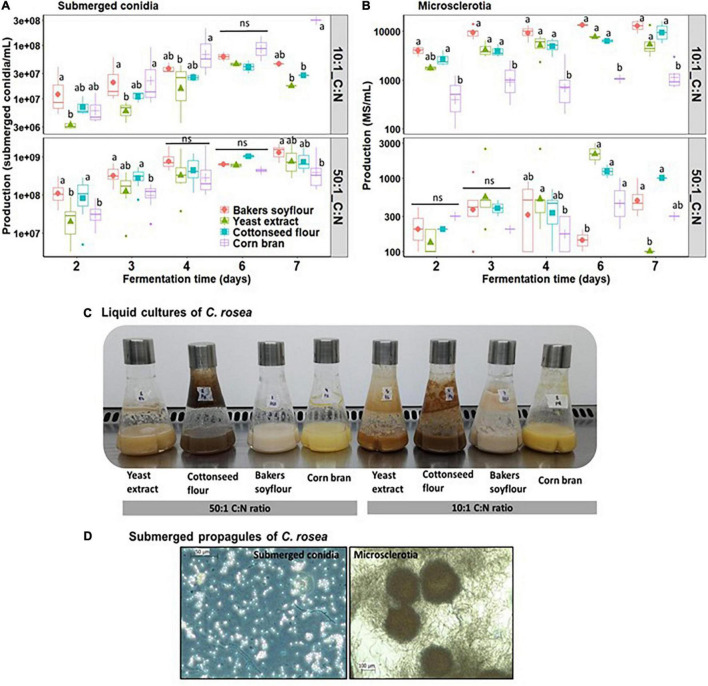
Effect of nitrogen interaction and C:N ratio on the production kinetics of submerged conidia **(A)** and MS **(B)** of *C. rosea* (strain CMAA1284) under submerged liquid culture in shake flasks. **(C)** Liquid cultures of *C. rosea* cultivated in shake flasks after 7 days of cultivation. **(D)** Microphotographs of submerged conidia and MS observed under light microscopy at 400× and 200× magnification, respectively. The means followed by the same letter within each time interval and C:N ratio (10:1 or 50:1) indicate that the nitrogen sources do not differ from each other (Tukey HSD, *p* < 0.05).

The submerged liquid fermentation carried out in shake flasks of *C. rosea* allowed the production of both submerged conidia and MS under appropriate C:N ratio, nitrogen source, and cultivation time. Notably, the interactions nitrogen source *vs.* C:N ratio and nitrogen source *vs.* cultivation time significantly influenced the production of submerged conidia, indicating that soybean meal as the nitrogen source with 50:1 C:N ratio on the seventh day of cultivation reached the maximum spore yield by liquid culture (*p* < 0.001, Supplementary Table S1). Clearly, the 50:1 C:N ratio had a remarkable impact on the increased yields of submerged conidia than 10:1 C:N ratio, in which the majority of liquid cultures attained spore production peak by day 7 of cultivation, although concentrations more than 1 × 10^9^ submerged conidia/ml were only achieved by day 7 of cultivation (Figure 2A). The nitrogen source also played a crucial role in the spore production, in which soybean meal followed by cottonseed flour and yeast extract stood out as the best, whilst corn bran exhibited the lowest performance. The highest submerged conidial production was achieved after 7 days of fermentation using soybean meal with 50:1 C:N ratio cultures that resulted in 1.41 × 10^9^/ml, followed by cottonseed flour corresponding to 1.03 × 10^9^/ml.

It is noteworthy that MS production was significantly influenced by three factors (*p* = 0.0067, Supplementary Table S1), the combination of soybean meal as the nitrogen source, 10:1 C:N ratio and 6 days of cultivation, resulting in a yield of 1.35 × 10^4^/ml by submerged cultures of *C. rosea*. However, from day 4 onward, the amount of MS did not significantly increase with longer cultivation times. Interestingly, yeast extract was the second-best nitrogen source that yielded 1.34 × 10^4^ MS/ml after 7 days of fermentation. With 50:1 C:N ratio, liquid cultures attained yields less than 3 × 10^3^/ml, whilst the opposite was found with higher nitrogen content imposed by lower C:N ratio that boosted MS yields over 1 × 10^4^/ml, regardless of nitrogen source and cultivation time (Figure 2B). Among cultures grown under 10:1 C:N ratio, the production peak of MS during fungal growth was reached between days 3 and 4 of cultivation, regardless of nitrogen source. Notably, the best nitrogen sources at 10:1 C:N ratio to produce MS followed the order listed as follows: Soybean meal, cottonseed flour, yeast extract, and the minor impact was attributed to the corn bran.

Overall, at a fixed amount of high carbon content (36 g/L), the C:N ratio clearly showed to dramatically affect propagule formation and production by submerged cultures of *C. rosea*, as higher C:N ratio favored the production of submerged conidia, whereas lower C:N ratio was preferred for MS development. This indicates that higher nitrogen content was crucial for fungal vegetative growth that leads to optimal MS production (Figure 2A).

All submerged cultures of *C. rosea* grown with 10:1 C:N ratio had a thicker aspect due to the higher nitrogen content promoting more vegetative growth and increased MS production (Figure 2C). The microsclerotia (MS) were typically a thread of mycelial aggregates usually larger than 50 μm and were dark pigmented (Figure 2D). In contrast, liquid cultures with 50:1 C:N ratio portrayed more liquid aspect or less viscosity due to less mycelial biomass and showed an increased production of submerged conidia of variable sizes (range of 3.86 to 8.21 μm in length and 2.89 to 4.71 μm in width), which were formed by conidiogenous cells known as phialides (Figure 2D).

### Small-Scale Fermentation in a Benchtop Bioreactor

To expand the production scale of *C. rosea*, the fermentation was carried out in an automated benchtop bioreactor, using the medium that contained soybean meal 0.84% (w/v), C:N ratio of 50:1, and initial pH of 6.0 (medium M3, Table 1), as it provided the greatest yield of submerged conidia under liquid culture in shake flasks.

**FIGURE 3:**
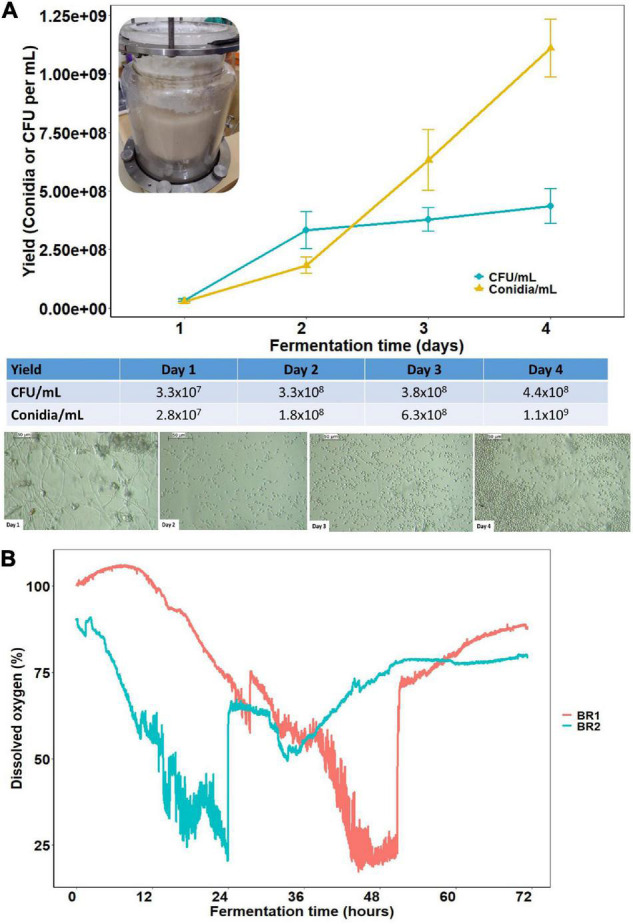
Batch submerged liquid fermentation of *C. rosea* (strain CMAA1284) carried out in a 3-L benchtop bioreactor using a medium containing dextrose monohydrate, soybean meal and adjusted to 50:1 C:N ratio, 36 g carbon/L and at 28°C. Evolution of submerged *C. rosea* submerged conidial production during the first, second, third, and fourth day of fermentation. **(A)** The yield of submerged conidia and total viable propagules expressed in CFU/ml, and a sequence of microphotographs of submerged conidia production over time. **(B)** Typical patterns of dissolved oxygen evolution during liquid culture growth of *C. rosea* in different fermentation batches.

Among the six fermentation batches performed, the pH initially set to 6.0 slightly dropped to 5.4–5.8 during the entire fermentation course (Table 2). To maintain the dissolved oxygen level above 15% throughout the fermentation course, the agitation speed increased from 400 to 700 rpm at the last day (day 4), in conjunction with the increase of sparged air (gas-flow) that varied from 1.0 to 2.0 L/min. Liquid cultures of *C. rosea* conducted in bioreactors depicted similar trends of DO level, although some variation was noted between these DO profiles across production batches with two typical patterns described in Figure 3B. As for the fermentation parameters, there was a rapid decrease in oxygen availability especially on the first day of cultivation across four fermentation batches, with oxygen consumption being more pronounced within 24 h post-inoculation. After 48 h of cultivation, it was noticed the dissolved oxygen level went back up to 50%, which also reflected a decrease in the specific growth rate from day 3 to day 4 (Table 2). Nevertheless, the concentration of the submerged conidia increased over time with a peak at day 4 corresponding to 1.1 × 10^9^/ml (Table 2 and Figure 3A). In turn, the number of total viable propagules (CFU) slightly increased from day 2 to day 4, resulting in a maximum yield of 4.35 × 10^8^ CFU/ml (Table 2 and Figure 3A). The variation in production of submerged conidia and total viable propagules from batch to batch also occurred and is described in Table 2. The microscopic observations also confirmed the progress of submerged conidial production during the fermentation course, as notably by day 4 depicting the highest conidial density.

**TABLE 2:** Submerged liquid fermentation parameters during *C. rosea* (strain CMAA1284) growth in a 3-L benchtop bioreactor across six independent batches (constant temperature set to 28°C).

Parameter	Fermentation course				
	Initial	Day 1	Day 2	Day 3	Day 4
Agitation speed (rpm)	400–450	500–550	550–600	600–650	650–700
Gas-flow (L/min)	1.0	1.5	1.5	2.0	2.0
pH	6.0	5.6 (5.5–5.9)	5.4 (4.7–5.9)	5.6 (5.2–6.1)	5.8 (5.3–6.1)
μ (1/h)^†^	–	0.065 (0.03–0.10)	0.073 (0.06–0.09)	0.065 (0.05–0.07)	0.056 (0.05–0.06)
CFU/ml	5 × 10^6^	3.33 × 10^7^ (2.0–4.0 × 10^7^)	3.32 × 10^8^ (1.6–6.0 × 10^8^)	3.77 × 10^8^ (2.0–5.4 × 10^8^)	4.35 × 10^8^ (2.8–6.0 × 10^8^)
Spores/ml	5 × 10^6^	2.77 × 10^7^ (2.0–4.0 × 10^7^)	1.82 × 10^8^ (2.0–4.0 × 10^7^)	6.7 × 10^8^ (2.0–10.0 × 10^8^)	1.11 × 10^9^ (0.75–1.5 × 10^9^)
Spores/g dried granules^§^	–	–	–	–	3.4 × 10^10^ (1.5–5.5 × 10^9^)
CFU/g dried granules^†^	–	–	–	–	2.0 × 10^9^ (0.6–3.2 × 10^9^)
Final moisture (%) of dried microgranules	–	–	–	–	2.93 (2.6–3.6)

*^†^Specific growth rate with initial time at 0 h and initial concentration set to 5 × 10^6^ submerged conidia/ml. ^§^Spore production (sporogenesis) of air-dried granular fungal biomass upon rehydration on water–agar medium after 7 days of incubation. ^†^CFU per gram of air-dried microgranules.*

The number of viable propagules (mostly submerged conidia) in the air-dried microgranular formulation of *C. rosea* was estimated to contain 2.0 × 10^9^ CFU/g. Since we used the M3 medium in the bioreactor studies, this growth environment stalled the formation of MS. At last, the air-dried microgranular formulation upon 24 h rehydration rendered 100% myceliogenic germination, followed by a conidial production of 3.4 × 10^10^ conidia/g microgranules at day 7 post-incubation, indicating a large number of spores released by this fungal formulation.

### Efficacy of Submerged *Clonostachys rosea* Propagules in the Inhibition of Sclerotial Germination of *Sclerotinia sclerotiorum* and Efficacy Against Whitefly Nymphs

All *C. rosea* propagules tested (i.e., aerial conidia, submerged conidia, and MS) against *S. sclerotiorum* parasitized 100% of sclerotia, even after surface sterilization of such structures, as noted by unchanged color of the Neon-S medium; whereas the untreated control exhibited all sclerotia viable as noted by the presence of profuse myceliogenic germination and color change from purple to yellowish of the Neon-S medium, which is indicative of oxalic acid production by the pathogen (Supplementary Figure S4). Furthermore, all non-viable sclerotia exhibited a profuse outgrowth of *C. rosea*, which strongly suggests that this bioagent internally colonized and parasitized sclerotia, regardless of its propagule type tested as inoculum (Supplementary Figure S4). These results indicate that *C. rosea* propagules (aerial conidia, submerged conidia, and MS), derived from either liquid or solid fermentation, were highly effective in constraining the myceliogenic germination of *S. sclerotiorum* sclerotia.

**FIGURE 4:**
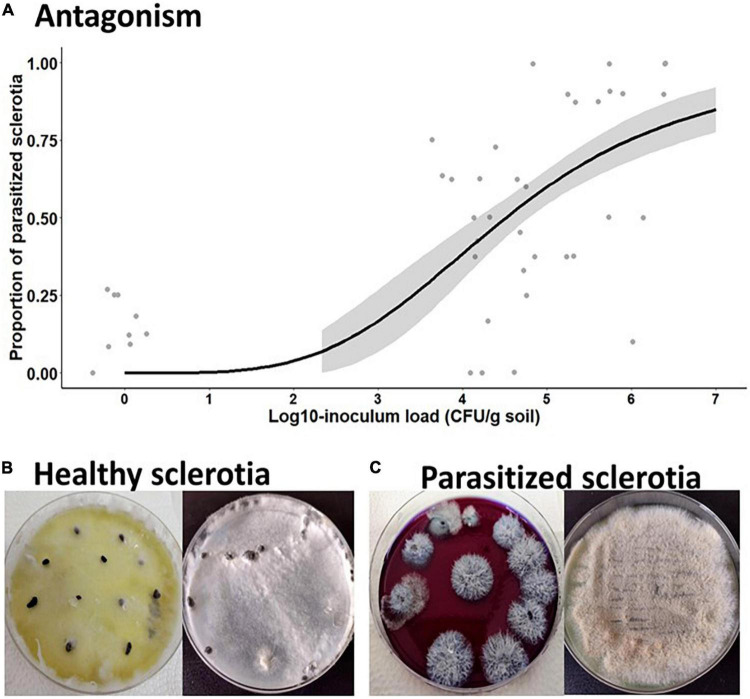
Antagonism of *C. rosea* (strain CMAA1284) submerged conidia over sclerotia of *S. sclerotiorum* after soil treatment. **(A)** Relationship of parasitized sclerotia with inoculum load of *C. rosea* tested in sterile soil substrate. **(B)** Healthy non-parasitized sclerotia showing color change in the Neon medium to yellowish due to synthesis of oxalic acid and recovery of pure colony of *S. sclerotiorum* on PDA medium. **(C)** Parasitized sclerotia showing no color change in the Neon medium, indicating they were not viable, as they showed outgrowth of *C. rosea* confirmed by plating the fungus on PDA.

In the follow-up experiment, there was a significant effect of *C. rosea* inoculum rate on sclerotial parasitism of *S. sclerotiorum* (χ^2^ = 33.98, *p* < 0.0001), in which all fungal concentrations differed from the untreated control group and induced sclerotial parasitism from 44.4 to 80.7% in a proportional manner (Supplementary Figure S5). It is noteworthy that the highest inoculum rate of *C. rosea* (1 × 10^6^ CFU/g of soil) caused the strongest reduction in sclerotial viability. The relation of sclerotial parasitism rate with the inoculum load of *C. rosea* applied to the soil arena was properly described with a two-parameter binomial log-logistic model (Figure 4A), in which the concentration required to suppress or parasitize 50% of the sclerotia was estimated to be 3.23 × 10^4^ CFU/g of soil (Table 3).

**TABLE 3:** Modeling concentration–parasitism relationship and estimation of median lethal concentration (LC_50_) of *C. rosea* (strain CMAA1284) submerged conidia against sclerotia of *S. sclerotiorum* (strain CMAA1105) under the controlled environmental conditions (14 days incubation, 25 ± 1°C with 12:12 h photoperiod).

Soil treatment	Model parameters (±SE)^†^		LC_50_ (×10^4^ CFU/g soil)	95% Confidence limits (×10^4^ CFU/g soil)	
	*E*	*b (slope)*		Lower	Upper
Air-dried submerged conidia	4.51	–3.92	3.23	1.66	6.29

*^†^Model equation: $y=\frac{1}{(1+exp\left(b\left(log\left(x\right)-log\left(e\right)\right)\right)}$ where y = proportion of sclerotial parasitism, b = slope, e = inflection point, x = inoculum load (CFU/g of soil).*

The healthy and viable *Sclerotinia* sclerotia from untreated control group were confirmed by the yellowish color produced by the Neon medium due to a reaction with oxalic acid secreted by the fungus (Figure 4B), whereas the submerged conidia of *C. rosea* were able to inhibit the myceliogenic germination of *S. sclerotiorum* sclerotia by impeding the color change in the Neon medium that remained purple (Figure 4C).

**FIGURE 5:**
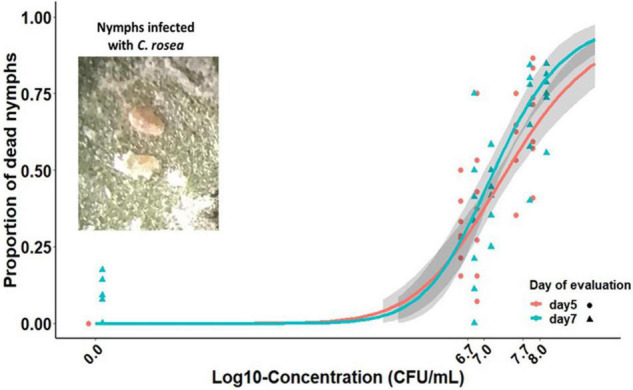
Susceptibility of the first to second whitefly instar nymphs (*B. tabaci* biotype B) to submerged conidia of *C. rosea* (strain CMAA1284) after 5 and 7 days of post-spraying. Typical dead nymphs due to infection of *C. rosea* are shown in the top left corner image. Solid lines represent fitted log-logistic curves describing the mortality and log_10_-concentration relationship, while symbols are data observations.

The insect bioassay revealed that *C. rosea* air-dried submerged conidia were pathogenic to early whitefly nymphs. The whitefly mortality significantly increased with incubation time and fungal concentration tested (interaction time × concentration: χ^2^ = 10.14, *p* = 0.0015), resulting in a range of 31–67.5% and 33–76.2% dead nymphs after 5 and 7 days of exposure, respectively. The two concentration-dependent mortality curves were different from each other (χ^2^ = 6.0, *p* = 0.0497), indicating that the whitefly mortality was relatively higher by day 7 than day 5 of evaluation, particularly when nymphs were exposed to higher fungal inoculum rates (Figure 5).

With respect to the median lethal concentrations and the mortality curve slopes, the whitefly mortality had similar slopes but differed in terms of LC_50_ values attained by different evaluation days, in which day 7 resulted in lower LC_50_ than day 5 (Table 4). In this line, the susceptibility of the whitefly nymphs to the *C. rosea* submerged conidia increased with time exposure, which resulted in a reduction of LC_50_ by 42.3% from day 5 to day 7 post-spraying (*p* = 0.048, Table 4). The dead nymphs due to infection with *C. rosea* usually exhibited symptoms of shriveled and orange-like appearance and signs of mycelial mats growing out of insect cadavers (Supplementary Figure S6).

**TABLE 4:** Modeling concentration-mortality relationship and estimation of median lethal concentration (LC_50_) at different exposure times after spraying *C. rosea* (strain CMAA1284) submerged conidia onto early whitefly nymphs (from first to second instars) on bean leaves.

Assessment post-spraying	Model parameters (±SE)^†^		LC_50_ (× 10^7^ CFU/ml)^§^	95% Confidence limits (×10^7^ CFU/ml)	
	*e*	*b (slope)* ^‡^		Lower	Upper
Day 5	7.41	–8.89^a^	2.6^a^	1.76	3.83
Day 7	7.18	–11.27^a^	1.5^b^	1.08	2.17

*^†^Model equation: $y=\frac{1}{(1+exp\left(b\left(log\left(x\right)-log\left(e\right)\right)\right)}$ where y = proportion of whitefly mortality, b = slope, e = inflection point, x = CFU concentration. ^‡^Slope comparison based on t-Student test: t = 1.07, p = 0.285 (not significant indicated by the same letters). ^§^Comparison between median lethal concentration values based on linear contrast: z = -1.977, p = 0.048 (statistically significant indicated by different letters).*

## Discussion

Our studies showed that *C. rosea* propagules can be mass produced in solid-state and liquid culture fermentation, and the resulting inoculum exhibited pathogenicity against two key soybean pests, *S. sclerotiorum* sclerotia and whitefly nymphs (*B. tabaci*) under laboratory conditions (Tables 3, 4 and Figures 4, 5). First, we demonstrate the impact of pre-culture (inoculum type) on conidial production *via* the solid-state fermentation and determine the feasibility of using liquid pre-culture for further inoculation of rice grains to shorten the cultivation time and to boost sporulation. This production method is known as two-stage or biphasic fermentation. The biphasic fermentation process for conidial production of numerous ascomycete fungal biocontrol agents is widely adopted around the world (Li et al., 2010; Mascarin and Jaronski, 2016).

In this work, by employing a two-stage fermentation process for *C. rosea*, we were able to achieve a maximum yield of 1.1 × 10^9^ conidia/gDM rice after 7 days of cultivation using parboiled rice as substrate. Various types of rice (white rice, parboiled rice, and broken rice) serve as both growth matrix and carbon and energy source in solid-state fermentation of many fungi used as biopesticides (Li et al., 2010; Mascarin et al., 2019; Santos P. S. et al., 2021). Nonetheless, further research on selecting agro-industrial wastes for *C. rosea* production by solid-state fermentation is needed to improve yields, shorten cultivation time, and reduce costs ensuring spore quality.

Relative yield rates are important when evaluating the mass production of the microbial biopesticides. Despite the singularities embedded in each protocol adopted, we noticed that the previous works attained higher yields than our current work, but reported longer cultivation times to achieve maximum conidial production, although the genetics of the strain could also account for these discrepancies. Viccini et al. (2007), for instance, observed a maximum production of 3.4 × 10^9^ conidia/gDM of *C. rosea* grown on the white rice grains (initial moisture content of 46%) and incubated the fungus for 60 days in Erlenmeyer flasks, while Zhang et al. (2013, 2015) reported yields in the range from 3.36 × 10^10^– to 3.50 × 10^10^ conidia/gDM in 11 days using a mixture of wheat bran and maize meal (3:1 w/w) in a horizontal tray reactor. Other studies reported production yields ranging from 1.1 × 10^8^ to 1.87 × 10^9^ conidia/gDM when the same Brazilian strain of *C. rosea* was grown for 15–30 days on moistened rice grains in polypropylene bags (Viccini et al., 2007; Carvalho et al., 2018). We noticed that *C. rosea* strain in our study takes considerably longer to fully colonize and sporulate the substrate when grown on rice inside polypropylene bags than when cultivated in Erlenmeyer flasks. This could be related to differences of surface–volume ratio, heat mass transfer, and oxygen supply in each type of reactor. The cost of solid-state fermentation for conidia production of *Trichoderma* and *Clonostachys* in Brazil has been estimated to be USD 7.07 to 14.67 for each kg of rice, and this represents an economical constraint for the large-scale production of *C. rosea* by solid-state fermentation.

In our study with liquid fermentation, we addressed the effects of nutritional environment modifications by altering C:N ratio and organic complex nitrogen source in submerged cultures of *C. rosea* for the production of conidia and MS. Furthermore, we also demonstrated that submerged conidia of *C. rosea* were enhanced when cultivated in a laboratory benchtop bioreactor with a controlled aeration rate which maintains a dissolved oxygen supply above 15% throughout the fermentation course. The bench-scale bioreactor is needed as a first step to assess the full-scale fungal bioprotectant production. Our results also indicated that submerged conidia remain viable and active when formulated as air-dried microgranules, displaying bioactivity against the whitefly (*B. tabaci* biotype B) nymphs and the sclerotia of *S. sclerotiorum* under laboratory conditions. Accordingly, these results expand the utility of *C. rosea* as a versatile and environmentally friendly biocontrol agent against multiple target hosts.

The cost of production is key when optimizing liquid culture media. Here, we found inexpensive nitrogen sources for submerged cultivation of *C. rosea*, which also led to maximum production of submerged conidia and MS, depending on the C:N ratio (Tables 1, 2 and Figures 2, 3). The nitrogen source and C:N ratio play a significant role in defining the production of submerged conidia or MS by submerged cultures of *C. rosea* (Figure 2). Corroborating our findings, Kobori et al. (2015) also noted the effects of the C:N ratio, the total carbon content, the carbon and nitrogen sources on the formation and the yield of submerged conidia and MS of *Trichoderma harzianum* strain T22 grown in submerged cultures. Although there was a preferred nitrogen source that sustained a better growth in the submerged culture, *C. rosea* was able to utilize and produce propagules when cultivated with all organic complex protein sources tested in this study. According to the multiple lifestyles of *C. rosea*, spanning saprophyte, endophyte, and mycoparasite, it is expected that this fungus would harbor a plethora of enzymes that allows exploitation of different complex carbon and nitrogen sources. The carbon content in *C. rosea* submerged cultures was set to 36 g/L, and supported higher sporulation than when grown with lower carbon titer, as previously reported for other filamentous fungi (Jackson and Jaronski, 2009; Kobori et al., 2015).

The main source of carbon was derived from dextrose monohydrate at a cost of USD 0.80/kg (Manufacturer’s information – Ingredion, Mogi Guaçu, SP, Brazil), while cottonseed flour (Pharmamedia^®^) and soybean meal (Baker’s soyflour^®^) were estimated at USD 2.70/kg and USD 0.53/kg, respectively (Manufacturer’s information – ADM, United States). The nitrogen source in the submerged culture is one of the most expensive medium constituents, and thus it justifies the search and selection of more suitable and inexpensive protein sources, especially those derived from agro-industrial residues or by-products, which are greatly desirable when designing media formulation for fungal biocontrol agents in a cost-effective way (Kampen, 2014). Particularly, the production of *C. rosea* submerged conidia using medium M3 (Table 1) with a benchtop bioreactor provided an estimated cost of USD 0.08/L, which is much more economically viable due to its shorter cultivation time (4 days instead of 7 days) when compared to the solid-state fermentation using parboiled rice.

The combination of the soybean meal, a high carbon content, and a low C:N ratio is required for a maximum production of MS of *C. rosea* by submerged liquid culture, resulting in 1.35 × 10^4^ MS/ml within 6 days. To the best of our knowledge, this is the first report of MS being produced during submerged liquid culture of *C. rosea*. Although first described here for *C. rosea*, previous studies have shown that the proper combination of nitrogen source, high carbon content, and lower C:N ratio induces the formation of relatively high numbers of MS by liquid cultures of *Metarhizium* spp. (Jackson and Jaronski, 2009), *Trichoderma* spp. (Kobori et al., 2015; Jackson et al., 2016), *Mycoleptodiscus terrestris* (Shearer and Jackson, 2006), *Colletotrichum truncatum* (Jackson and Schisler, 1995), and *Beauveria* spp. (Villamizar et al., 2018). For instance, *Beauveria* spp. can deliver MS yields in the order of 10^3^ MS/ml after 10 days of cultivation (Wang et al., 2011; Villamizar et al., 2018). Those values are lower than those reached for MS production with other fungal species (>10^4^ MS/ml) in shorter fermentation times (3–5 days) (Jackson and Jaronski, 2009; Mascarin et al., 2014; Song et al., 2014, 2016; Kobori et al., 2015).

Since *C. rosea* is an excellent mycoparasite of many fungal plant pathogens (Sun et al., 2020), it would be of much interest to develop a more resilient propagule, such as a resting structure-like microsclerotium, to be applied directly to soil where this fungus can survive saprophytically and suppress the development of other plant pathogenic fungi. As such, *C. rosea* MS can survive under low moisture and eventually produce aerial conidia after myceliogenic germination induced by adequate soil moisture. All of these attributes suggest that MS can be incorporated into dry granules as a new sustained release strategy to control soilborne plant pathogenic fungi, such as *S. sclerotiorum* and *Botrytis cinerea*. Moreover, the microsclerotium could potentially be employed in the seed treatment and would probably exhibit longer storage stability than submerged conidia, but this hypothesis remains to be investigated.

The benefits around the submerged fermentation method performed in stirred-tank bioreactors include shortening the cultivation time accompanied by high yields in the production of the desired fungal propagule. In this sense, we validated and improved the production of submerged conidia of *C. rosea* using a laboratory benchtop bioreactor supplying proper oxygenation to sustain rapid growth. The culture pH during the fermentation course does not require control, which facilitates the cultivation process. This setup led to yields of up to 1.2 × 10^9^ submerged conidia/ml within just 4 days of cultivation. Hence, this bioprocess provides the highest concentration of submerged conidia in shortest time ever attained by *C. rosea*, when compared with earlier liquid fermentation studies which reported a maximum concentration in the range of 1.01 × 10^8^ to 3.3 × 10^8^ submerged conidia/ml after 7 days of fermentation (Sun et al., 2013; Carvalho et al., 2018). Although chlamydospores were reported by Sun et al. (2013), we have not found any formation of neither this resting spore nor blastospores in submerged cultures of *C. rosea*. In comparison to solid-state fermentation, the scale-up production of conidia by submerged liquid fermentation in stirred-tank fermenters requires much less physical space, manpower, and time, which are critical factors to consider for the cost-effective mass production of this fungal biopesticide.

The air-dried microgranular formulation obtained here with *C. rosea* produced by submerged fermentation, after optimal conditions for rehydration, produced 3 × 10^10^ conidia/g, being superior when compared to the spore production obtained with air-dried MS granules of *Beauveria* spp., *Metarhizium* spp., and *T. harzianum* (Jackson and Jaronski, 2009; Behle and Jackson, 2014; Mascarin et al., 2014; Kobori et al., 2015; Villamizar et al., 2018). This indeed opens a new venue for exploring submerged fermentation as an efficient method for mass production of *C. rosea* submerged propagules and as a possible alternative to the solid-state fermentation process.

The potential of *C. rosea* as a biological control agent has received great attention owing to its broad spectrum of target hosts, such as plant pathogens and insect pests (Iqbal et al., 2018b). The versatility of *C. rosea* is attributed to the activation of multiple mechanisms, such as secreted cell-wall-degrading enzymes, production of secondary antifungal metabolites, and induction of plant defense systems (Iqbal et al., 2018a; Sun et al., 2020). Our results reveal that submerged conidia and MS were able to inhibit 100% sclerotial germination of *S. sclerotiorum*, which showed the same antagonistic effect as aerial conidia of *C. rosea* produced by solid-state fermentation. Furthermore, we observed that *C. rosea* is able to parasitize and kill sclerotia, completely halting their germination. By testing different inoculum concentrations, we found a pronounced antagonistic effect by air-dried microgranules formulation of *C. rosea* submerged conidia (1 × 10^6^ CFU/g soil) in inhibiting myceliogenic germination of *S. sclerotiorum*, which resulted in 88.0% parasitism of those sclerotia. Corroborating this result, Wu et al. (2018) verified that a dry flowable formulation of *C. rosea* conidia produced by submerged fermentation was effective against *S. sclerotiorum* when sprayed on the cucumber seedlings, with a control efficiency of 88.3%, albeit these authors did not mention the inoculum concentration tested.

The necrotrophic mycoparasitic lifestyle exhibited by *C. rosea* and other mycoparasitic fungi of the order Hypocreales (e.g., *Trichoderma* spp., *Tolypocladium ophioglossoides*, *Escovopsis weberi*) is more destructive and often unspecialized than biotrophs (Karlsson et al., 2017; Sun et al., 2020). The previous studies described the mechanisms involved in the mycoparasitic relationship between *C. rosea* and *S. sclerotiorum* or other plant pathogenic fungi as well as plant parasitic nematodes by revealing a plethora of cell-wall-degrading enzymes in concert with toxic secondary metabolites, such as peptaibols (Rodríguez et al., 2011; Iqbal et al., 2018a,b; Wu et al., 2018; Sun et al., 2020). These mechanisms are also related to its host range and virulence degree. Therefore, it is reasonable to suggest that the mycotrophic behavior of the *C. rosea* strain CMAA1284 also relies on these mycoparasitism mechanisms to suppress sclerotial germination. For instance, Rodríguez et al. (2011) demonstrated that *C. rosea*, during the mycoparasitism on *S. sclerotiorum* mycelium, secretes peptaibols causing hyphal cell lysis and interrupting fungal growth. Therefore, the elucidation of the mechanisms involved in the parasitic behavior of *C. rosea* for its wide host range are among the key factors for understanding its different ecological strategies and for its development as an effective biocontrol agent.

The activity of submerged *C. rosea* conidia was demonstrated for the first time against the *B. tabaci* biotype B using young nymphs (Figure 5), with maximum mortality (i.e., 76.2% dead nymphs) observed 7 days after spraying *C. rosea* at a concentration of 1 × 10^7^ CFU/ml. Anwar et al. (2018) verified that the whitefly adults of *B. tabaci* at 6 days post-spraying with a *C. rosea* concentration at 4 × 10^8^ conidia/ml reached 23.54% mortality. Moreover, pathogenicity of *Clonostachys* species has been also reported against other insect pests, such as *Hypothenemus hampei* (Vega, 2008), leafhoppers (*Oncometopia tucumana* and *Sonesimia grossa*) (Toledo et al., 2006), *Delia radicum* (Razinger et al., 2014), and *Carpomya vesuviana* (Mahmoudi et al., 2018). Another advantage of *C. rosea* resides in its amenability in combined application with entomopathogenic fungi for biocontrol of insect pests and fungal pathogens (Kapongo et al., 2008). Also, *C. rosea* possesses a resourceful enzymatic arsenal, including proteases and chitinases, which may play a role in the infection process of several target hosts, including arthropods, nematodes, and plant pathogenic fungi (Zhao et al., 2005; Li et al., 2006; Gan et al., 2007). The annotated genomes of different strains of *C. rosea* (Karlsson et al., 2015; Sun et al., 2015) have unveiled a plethora of biological-control related genes encoding many groups of lytic enzymes (e.g., glucanases, proteases, chitinases, and monooxygenases) and secondary metabolites (e.g., non-ribosomal peptide synthetases and polyketide synthases) that can play a role in its entomopathogenic strategy.

Although the submerged conidia numbers produced in our best culture conditions were up to 10^5^ higher than the MS production, both propagules had the same effectiveness in suppressing *S. sclerotiorum* sclerotia (Supplementary Figure S3). When targeting the whitefly nymphs, the submerged conidia seems to be the preferred type of propagule for spray application on aboveground plant parts, such as leaves where this insect inhabits. In contrast to that approach, air-dried MS require relatively high relative humidity within a certain period of time to resume myceliogenic germination and subsequently sporulate on a substrate, and that is critical for conidia production and further infection of the target insect (Rodrigues et al., 2021). When focusing on soil application, *C. rosea* microsclerotium would be most suitable due to its resistant nature and ability to withstand adverse environmental conditions, as in the case of *Metarhizium anisopliae* (Ascomycota: Clavicipitaceae) where its MS exhibited higher tolerance to UV-B radiation compared to aerial conidia (Santos T. R. et al., 2021). In support of this, MS of other biocontrol filamentous fungi have been shown to control their target pests when MS preparations are delivered to the soil, and upon the appropriate moisture, they undergo myceliogenic germination followed by sporogenesis (Schisler and Jackson, 1996; Jackson and Jaronski, 2009; Kobori et al., 2015; Villamizar et al., 2018; Marciano et al., 2021; Santos T. R. et al., 2021). The phenotypical plasticity of *C. rosea* to produce submerged conidia and MS allows us to use submerged conidia for aboveground applications and the MS in soil or seed treatment.

Regarding shelf life, it is of paramount importance to determine the survivability of submerged conidia and MS during refrigerated and non-refrigerated storage conditions. It is also imperative to investigate and compare these submerged propagules with traditional aerial conidia-based products prior to establishing submerged culture as the mainstream mass production process for *C. rosea* by industry. Future work will compare field efficacy between the submerged propagules and the aerial conidia. This would allow us to determine the best fungal propagule type for use as active ingredient in commercial formulations.

In summary, *C. rosea* is capable of forming submerged conidia and MS when grown in submerged culture under appropriate nutritional environment mediated by C:N ratio and nitrogen source. Our results indicate that the submerged conidia yields can be enhanced and rapidly produced in stirred-tank bioreactors. Moreover, submerged culture of *C. rosea* may be considered as an alternative to the traditional solid-state fermentation for its large-scale production. All these attributes indicate that our proposed microgranular formulation that contained *C. rosea* submerged conidia may provide an additional cost-effective tool for sustainable and environmentally friendly management of *B. tabaci* and *S. sclerotiorum*.

## Data Availability Statement

The raw data supporting the conclusions of this article will be made available by the authors, without undue reservation.

## Author Contributions

GM, NK, and WB contributed to conception and design of the study and performed the statistical experiments analysis and wrote the first draft of the manuscript. AS, GM, and TS performed the all experiments. GM and AS organized the database. GM, MM, NK, and WB wrote sections of the manuscript. All the authors contributed to manuscript revision, read, and approved the submitted version.

## Conflict of Interest

GM, AS, TS, MM, and WB was employed by Brazilian Agricultural Research Corporation. The remaining author declares that the research was conducted in the absence of any commercial or financial relationships that could be construed as a potential conflict of interest.

## Publisher’s Note

All claims expressed in this article are solely those of the authors and do not necessarily represent those of their affiliated organizations, or those of the publisher, the editors and the reviewers. Any product that may be evaluated in this article, or claim that may be made by its manufacturer, is not guaranteed or endorsed by the publisher.

## Footnotes

1
https://cran.r-project.org/
