# Supplementary material for: Clonostachys rosea: Production by Submerged Culture and Bioactivity Against Sclerotinia sclerotiorum and Bemisia tabaci
Source: Front Microbiol. 2022 May 6;13:851000. doi: 10.3389/fmicb.2022.851000 (PMC9120755; doi:10.3389/fmicb.2022.851000)
Supplement: Supplementary file 1 [file Data_Sheet_1.pdf]

Supplementary Material

Supplementary Figures

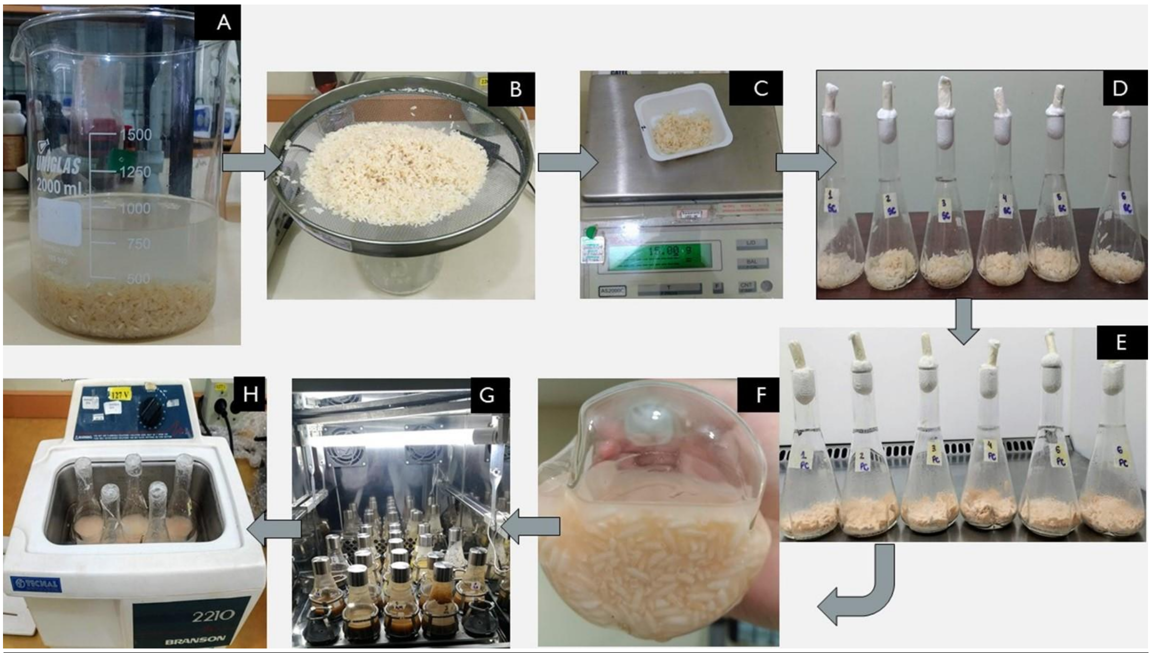

Figure S1. Flowchart depicting the procedure for solid-state fermentation employed to produce aerial conidia of *Clonostachys rosea* (strain CMAA1284) on autoclaved moistened rice grains. A – B) Moistening for 60 min and later on filtering out water from the rice grains. C – D) Weighing 15 g of moistened rice grains and transferring to 125-mL baffled Erlenmeyer flasks capped with cotton stoppers. E) Fully colonized rice mass with *C. rosea* after 7 days incubation at 25 ± 1 ºC and 12 h photophase. F – G) Addition of 50 mL of surfactant solution (0.05% Break Thru® MSO, Evonik®, Essen, Germany) to each flask, placed in a rotary incubator shaker for 30 min. H) Flasks were then taken to a ultrasound water bath for 5 min in order to promote the detachment of conidia from the substrate prior to proceeding with counts on a Neaubauer chamber.

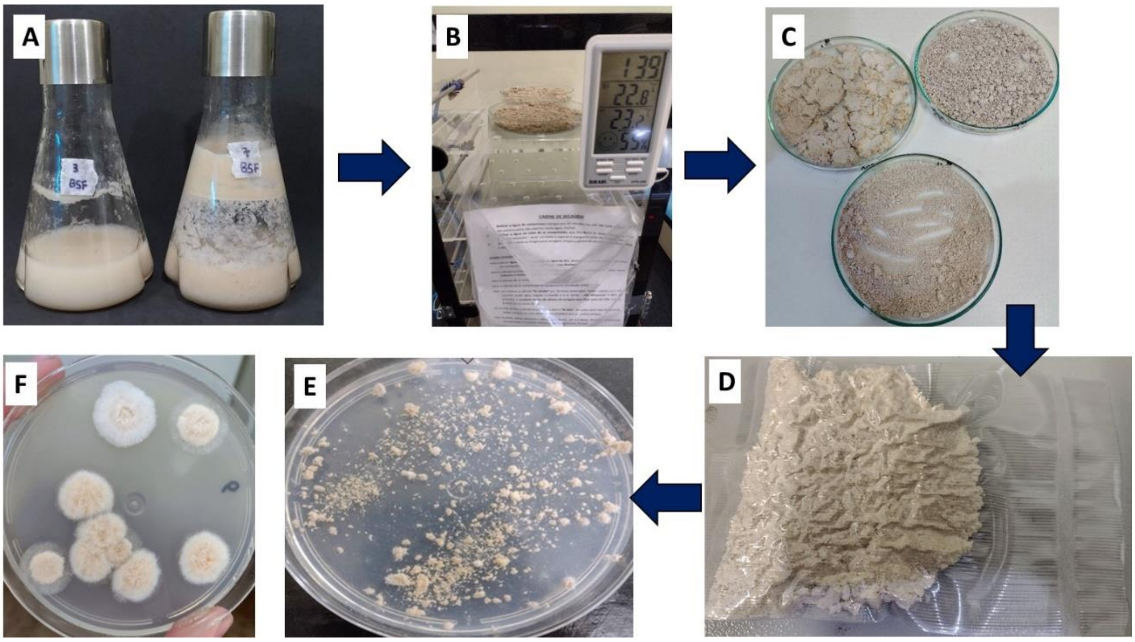

Figure S2. Flowchart illustrating schematically the formulation preparation of either submerged conidia or microsclerotia produced by liquid-culture grown *Clonostachys rosea* (strain CMAA1284). A) Liquid-grown *C. rosea* in shake flasks for conidia and microsclerotia production. B) After mixing whole fungal biomass with 5% (w/v) diatomaceous earth (FE), the fungus-DE mixtures were dehydrated inside a horizontal air drying chamber for about 14-16 h at 22 ± 2 °C. C) Physical aspect of the granular fungal preparations after air drying and hereafter referred to as air-dried fungal granules. D) Vacuum packaged air-dried fungal granules and cold stored until use in experiments. E) Assessment of sporogenic production on fungal granules upon rehydration after 7 days of incubation at 25 ± 1 ºC and 12 h photophase. F) Colony forming units (CFU) determined after plating a serially diluted suspension made with fungal granules to assess cell viability.

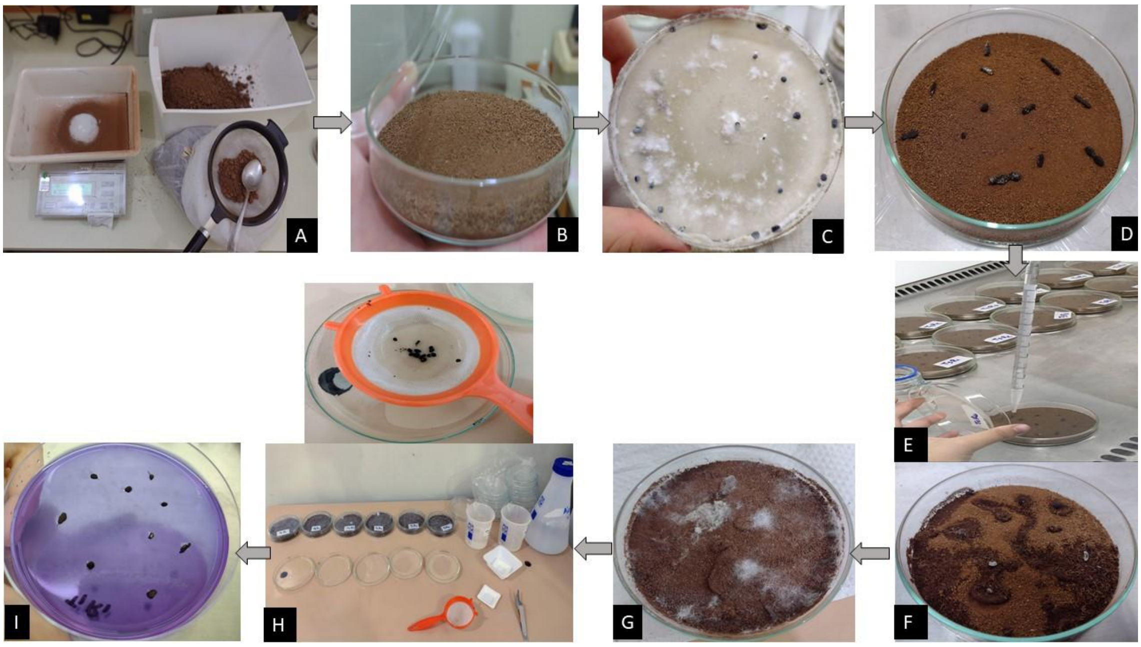

Figure S3. Flowchart depicting the procedure carried out for assessment of *in vitro* antagonism of *Clonostachys rosea* (strain CMAA1284) on sclerotia of *Sclerotinia sclerotiorum* (strain CMAA1105) using sterile soil as substrate. A – B) Sterile soil weighed and 30 g transferred to 90 x 15 cm Petri dish. C) Mature melanized sclerotia of *S. sclerotiorum* grown on PDA medium. D) Transfer and distribution of 12 sclerotia on the soil surface. E – F) Adding *C. rosea* propagule suspension (10.5 mL per plate) from different treatments (aerial conidia, submerged conidia and microsclerotia). G) White moldy cottonish aspect of sclerotia after 14 days in a growth chamber at 25 ± 1 °C with 12 h photophase, an indicative that there might have occurred either parasitism or the pathogen exhibited myceliogenic germination. H) Surface sterilization procedure for sclerotia retrieved from soil in the presence or absence of *C. rosea* treatment. I) Surface-sterilized sclerotia plated on Neon medium for assessment of sclerotia viability after 7 days of incubation at 25 ± 1 °C with 12 h photophase.

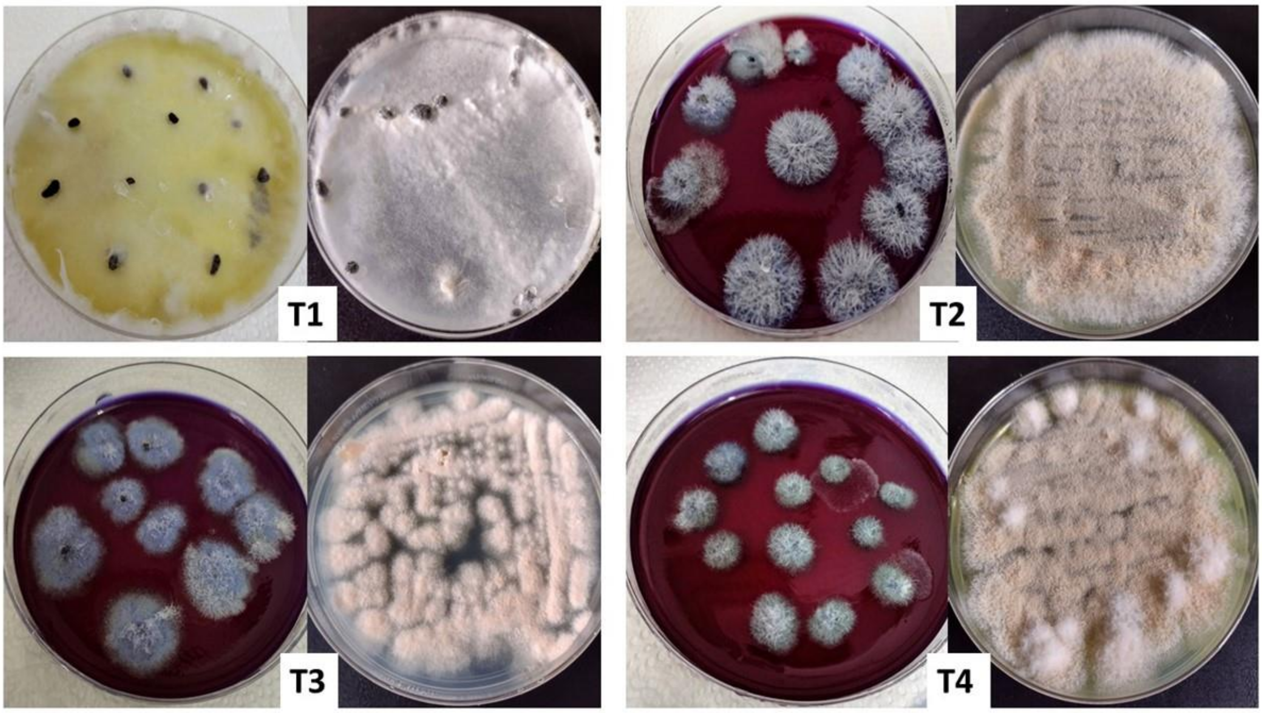

Figure S4. Mycoparasitism exerted by *Clonostachys rosea* (strain CMAA1284) impairing the myceliogenic germination of *Sclerotinia sclerotiorum* (strain CMAA1105) sclerotia, even after surface sterilization of sclerotia. Treatments are shown as: T1 (Control) – viable sclerotia presenting 100% normal germination and depicting color change of the Neon-S medium to yellowish as an indicative of oxalic acid production; T2 (*C. rosea* aerial conidia), T3 (*C. rosea* submerged conidia) and T4 (*C. rosea* microsclerotia) all showed 100% outgrowth and colonization of sclerotia, even after surface sterilization, with the absence in color change of the Neon-S medium, indicating that sclerotia were parasitized and killed by this bioagent, regardless of its propagule tested as inoculum.

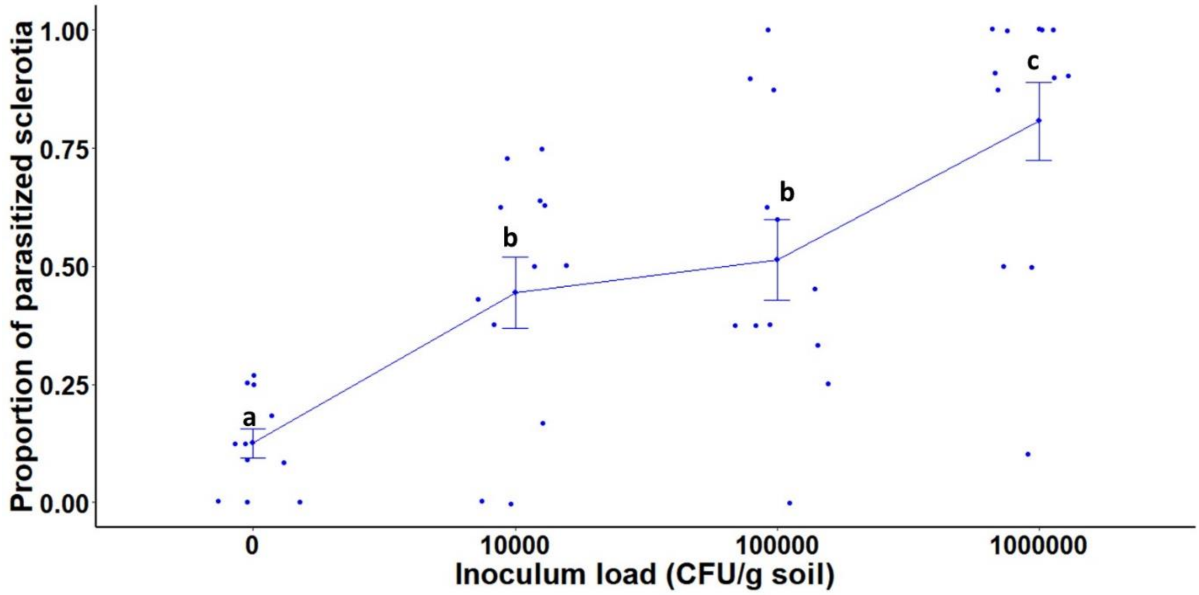

Figure S5. Effect of inoculum load (concentration of CFU per gram of soil) of *Clonostachys rosea* (strain CMAA1284) on the parasitism of sclerotia of *Sclerotinia sclerotiorum* (strain CMAA1105) under controlled environmental conditions (25 ± 1 °C with 12 h photophase). Lettering indicates significant differences between inoculum load (Tukey HSD, $p < 0.05$).

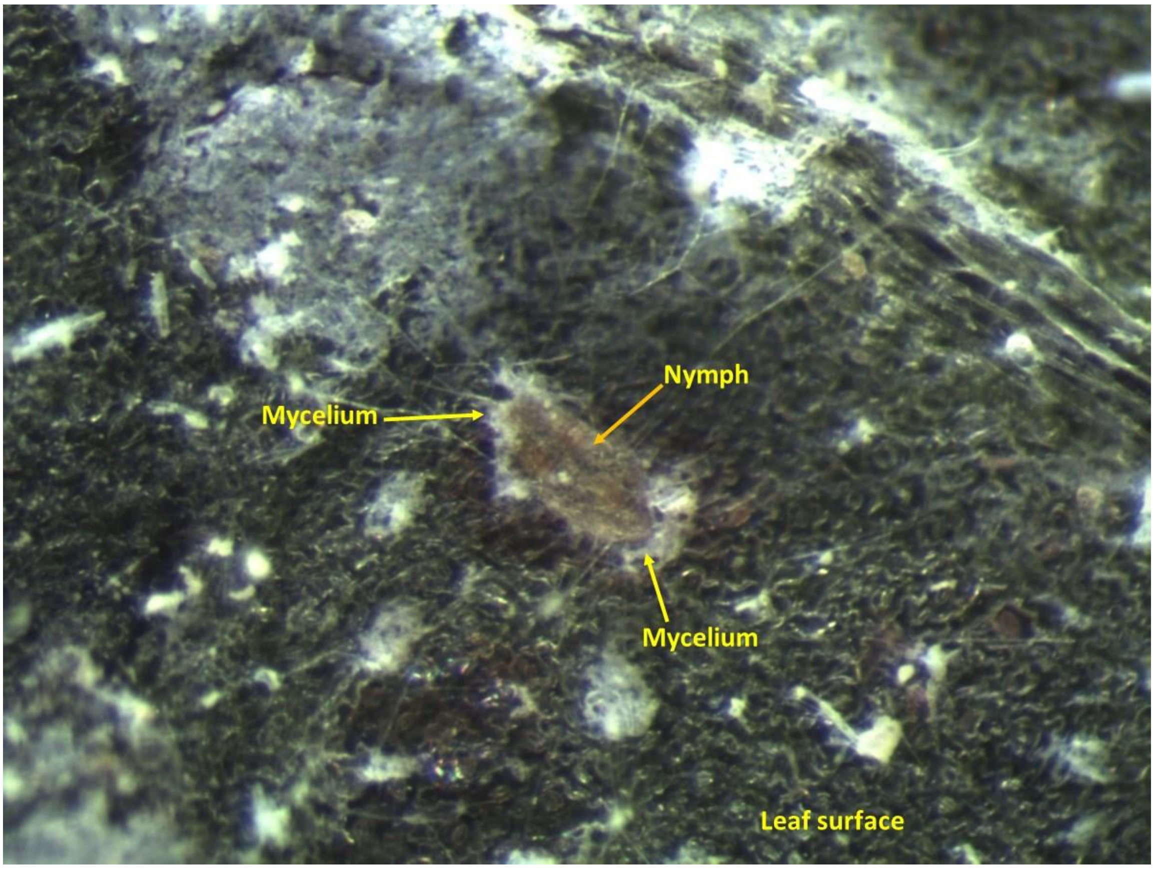

Figure S6. Stereomicroscopic photograph depicting a whitefly nymph (2nd instar) infected and colonized by *Clonostachys rosea* (strain CMAA1284) on a bean leaf surface. Arrows indicate the nymph with a shriveled, dehydrate, and orange-like appearance showing mycelial mats growing out the cadaver.

Supplementary Tables

Table S1. Deviance table (negative binomial model) for the interaction of nitrogen source, C:N ratio and fermentation day on the production of submerged conidia and microsclerotia of *Clonostachys rosea* (strain CMAA1284) under liquid culture conditions.

**Predictors**	**Submerged conidia**			**Microsclerotia**		
	χ^2	**df**	***p-value***	χ^2	**df**	***p-value***
Nitrogen source (A)	52.55	3	2.285e-11 ***	150.07	3	< 2.2e-16 ***
C:N ratio (B)	539.68	1	< 2.2e-16 ***	623.92	1	< 2.2e-16 *
Day (C)	450.96	4	< 2.2e-16 ***	67.86	4	6.434e-14 ***
A:B	41.58	3	4.914e-09 ***	43.09	3	2.345e-09 ***
A:C	28.05	12	0.0054 **	30.05	12	0.0027 **
B:C	4.96	4	0.290 ns	4.08	4	0.394 ns
A:B:C	10.09	12	0.607 ns	27.44	12	0.0067 **

Significant codes for *p-value*: 0 ‘***’, 0.001 ‘**’, 0.01 ‘*’, 0.05 ‘ns’.
